# Translational Fusion to Hmp Improves Heterologous Protein Expression

**DOI:** 10.3390/microorganisms10020358

**Published:** 2022-02-04

**Authors:** Xuanqing Wan, A. James Link, Mark P. Brynildsen

**Affiliations:** Department of Chemical and Biological Engineering, Princeton University, Princeton, NJ 08544, USA; xuanqing@princeton.edu (X.W.); ajlink@princeton.edu (A.J.L.)

**Keywords:** flavohemoglobin, N-terminal tag, bacterial hemoglobin, nitric oxide

## Abstract

Flavohemoglobins, which are widely distributed in prokaryotes and eukaryotes, play key roles in oxygen (O_2_) transport and nitric oxide (·NO) defense. Hmp is the flavohemoglobin of *Escherichia coli*, and here we report that the translational fusion of Hmp to the N-terminus of heterologous proteins increases their expression in *E. coli*. The effect required the fusion of the proteins, and was independent of both the O_2_-binding and catalytic activity of Hmp. Increased expression was at the translational level, likely to be downstream of initiation, and we observed that as little as the first 100 amino acids of Hmp were sufficient to boost protein production. These data demonstrate the potential of Hmp as an N-terminal fusion tag to increase protein yield, and suggest that the utility of bacterial hemoglobins to biotechnology goes beyond their O_2_ transport and ·NO detoxification capabilities.

## 1. Introduction

Flavohemoglobins are composed of globin domains, which can bind heme, fused to FAD- and NAD(P)-binding domains that facilitate electron transfer [1]. They are widely distributed in prokaryotes and eukaryotes and Hmp is the flavohemoglobin of *E.* *coli*, which is arguably the most studied [2]. While the main functions of mammalian globins, such as hemoglobin and myoglobin, are O_2_ transport and supply, the main function of Hmp has been attributed to nitric oxide (·NO) detoxification, where it uses O_2_ and reducing equivalents to convert ·NO to NO_3_^−^ [3,4]. Indeed, Hmp is the main ·NO detoxification enzyme for *E. coli* at dissolved O_2_ concentrations ranging from fully aerobic down to 1 µM [5]. Additional reactions Hmp has been found to catalyze include ·NO conversion to N_2_O under anaerobic conditions, and the reduction of O_2_ to O_2_^−^ in the absence of ·NO, which can be deleterious [6,7]. Hmp expression in *E. coli* is largely regulated by NsrR, which represses its transcription in the absence of ·NO, and thus, under normal growth, Hmp is present in cells at trace levels and is induced in response to nitrosative stress [8,9].

Beyond Hmp, numerous microbial hemoglobins have been implicated in ·NO detoxification [10]. However, it was their O_2_-binding capacity that inspired researchers to use them in high-density fermenters to serve as an O_2_ reservoir [11,12]. For example, Khosla and Bailey expressed *Vitreoscilla hemoglobin* (VHb) in *E. coli* from a plasmid and observed increased growth compared to the empty vector control [11], whereas later work with genomically-integrated VHb increased protein production under O_2_ limitations [12]. Bioprocess applications of VHb overexpression continued, and improvements to protein expression, overall fitness, and chemical production were observed in multiple microbes, including *E. coli* [13,14], *B. subtilis* [15], *P. aeruginosa* [16], *Streptomyces* sp. [17], *S. cerevisiae* [18], *P. pastoris* [19], and *S. cinnamonensis* [20]. Initial hypotheses about the mechanism behind these phenomena centered on the O_2_-binding capacity of VHb, and its ability to serve as an O_2_ source under O_2_-limited conditions, and thus increase the efficiency of metabolism [12,13,21,22,23]. Later, the ·NO detoxification capabilities of bacterial flavohemoglobins became apparent, which could protect respiratory terminal oxidases from inhibition by ·NO, and it was found that VHb directly binds to a component of cytochrome *bo*, which could also impact respiratory metabolism [21,24,25]. Indeed, while the exact mechanism remains incompletely defined [26], VHb has proven to be a valuable biotechnology tool for high-density, low-O_2_ fermentations [22,27].

Here, we describe our discovery that the fusion of Hmp to various heterologous proteins (super folder GFP (sfGFP), mCherry, bovine β-casein (β-csn)) significantly increases their expression in *E. coli*. The phenomenon required Hmp to be fused to the N-terminus of the target protein, it required at least the first 100 amino acids of Hmp, and it was not dependent on the catalytic activity or O_2_-binding ability of Hmp. Importantly, unlike the effect of VHb, the expression of Hmp as a non-fused protein failed to increase target protein production appreciably. Results provided here demonstrate the potential use of Hmp as a fusion tag, and suggest a novel mechanism by which bacterial hemoglobins can increase protein production in biotechnology.

## 2. Materials and Methods

### 2.1. Strains and Plasmids

Bacterial strains and plasmids used in this study are summarized in Supplemental Table S1 [28,29,30,31,32,33,[34,35,36]. *E. coli* K-12 MG1655 strain was used as wild-type. Plasmids were constructed with Q5 site-direct mutagenesis kit (New England Biolabs, Ipswich, MA, USA) or Hifi DNA assembly kit (New England Biolabs, Ipswich, MA, USA) using plasmids from previous studies, synthetic DNA inserts (Genewiz, South Plainfield, NJ, USA), or genomic DNA as templates. All plasmid sequences were confirmed with Sanger sequencing (Genewiz, South Plainfield, NJ, USA).

### 2.2. Growth Media and Chemicals

Components of growth media and antibiotics were purchased from Fisher Scientific (Pittsburgh, PA, USA), Sigma Aldrich (Milwaukee, WI, USA), or Thermo Fisher Scientific (Waltham, MA, USA) and isopropyl-β-d-thiogalactopyranoside (IPTG) was purchased from Gold Biotechnology (St. Louis, MO, USA). Growth media used in this study were LB broth and M9 minimal media. LB broth was made by dissolving pre-mixed powder (40/40/20 weight percent tryptone, NaCl, and yeast extract, respectively) in Milli-Q water (resistivity of 18.2 MΩ × cm) in a ratio of 25 g LB mix/L water, which was then autoclaved. Concentrated (5X) M9 minimal salts solution was made by dissolving 33.9 g/L Na_2_HPO_4_, 15 g/L KH_2_PO_4_, 5g/L NH_4_Cl, and 2.5 g/L NaCl in Milli-Q water, which was then autoclaved. To make M9 minimal media, Milli-Q water, 5× M9 minimal salts solution, CaCl_2_, MgSO_4_, and glucose were mixed to reach a final concentration of 1× M9 minimal salts, 0.1 mM CaCl_2_, 2 mM MgSO_4_, and 10 mM glucose, which was then sterilized with a 0.22 µm filter (Thermo Fisher Scientific, Waltham, MA, USA).

### 2.3. Fluorophore Expression Assay

Cells were grown aerobically from −80 °C frozen stock in 1 mL LB at 37 °C and 250 rpm for 4 h. Ten µL of the LB pre-culture was used to inoculate 1 mL of M9 minimal media at 37 °C and 250 rpm for overnight growth. The overnight culture was inoculated into 2 mL of fresh M9 media to an OD_600_ of 0.01, and grown until mid-exponential phase (OD_600_ = 0.2). At that point, cultures were induced with 1 mM IPTG when needed and transferred to 96-well black, clear-bottom plates (Corning Incorporated Inc., Corning, NY, USA) that were incubated at 37 °C and 250 rpm with the lid on. Ampicillin (100 μg/mL) or kanamycin (50 μg/mL) was added to cultures as needed for plasmid maintenance.

Cell concentration (OD_600_) and fluorescence (485/515 nm excitation/emission for sfGFP, or 580/610 nm excitation/emission for mCherry) were quantified using a SynergyTM H1 Hybrid Microplate Reader in 96-well black, clear-bottom plates (Corning Incorporated Inc., Corning, NY, USA). sfGFP/OD_600_ was reported after subtracting the background signal, which was taken as sfGFP/OD_600_ of wild-type carrying an empty pQE80L or pUA66 plasmid that was induced identically as samples. When the fluorescence signals were near the limit of detection for the plate reader (mCherry expressed by MO001 strain, and sfGFP expressed by pUA66 plasmid), measurements were conducted with flow cytometry instead. For flow cytometry, at each time point, 300 µL samples were collected, centrifuged at 15,000 rpm for 3 min, fixed with 4% (*w*/*v*) paraformaldehyde for 15–30 min, washed with PBS, and fluorescence was measured with LSRII flow cytometer (BD Biosciences, San Jose, CA, USA) with bandpass filters of 525/50 nm for sfGFP and 610/20 nm for mCherry. Median fluorescence readings (AU) from samples were used.

### 2.4. β-Galactosidase Assay

Δ*lacZYA* harboring pQE80L, pQE80-*hmp-lacZ*, and pQE80-*lacZ* were assayed for β-galactosidase activity. Cells were prepared as described in Section 2.3. The β-galactosidase assay was conducted following the protocol described by Miller [37], with minor modifications to adapt measurements for 96-well plates [38]. Briefly, upon reaching the exponential phase of growth, cells were induced with 1 mM IPTG. At each time point, 300 µL samples were collected, centrifuged at 15,000 rpm for 3 min, and washed with Z-buffer (60 mM Na_2_HPO_4_ · 7H_2_O, 40 mM NaH_2_PO_4_ · H_2_O, 10 mM KCl, 1 mM MgSO_4_ · 7H_2_O, 50 mM β-mercaptoethanol). OD_600_ was measured at that point, and then cells were permeabilized with 5 µL of 1% SDS and 10 µL chloroform, vortexed for 10 s, and stored on ice. After collection of all samples, and allowing the chloroform to settle to the bottom of the centrifuge tubes, permeabilized samples (1 µL for induced and 100 µL for uninduced) were transferred to flat-bottom 96-well plates (USA Scientific, Inc., Enfield, CT, USA). Z-buffer was added to each well of induced samples to reach 100 µL of total volume, and the reaction was initiated by the addition of 20 µL ONPG (4 µg/mL). After incubation at room temperature for an appropriate length of time (7 min for induced and 12 min for uninduced), the reaction was terminated by the addition of 50 µL of 1M Na_2_CO_3_. The absorption at 420 nm (OD_420_) and 550 nm (OD_550_) were measured, and the Miller units (MU) were calculated based on the formula below [37]:

$$\mathrm{Miller}\;\mathrm{Units}\;=\frac{1000\times \left({\mathrm{OD}}_{420}-1.75\;{\times \mathrm{OD}}_{550}\right)\;}{{T\times V\times \mathrm{OD}}_{600}\;}$$

where T is the time of the reaction in minutes, and V is the volume of culture used in the assay in mL. We note that the procedure was modified slightly between induced and uninduced samples because of the large difference in signal between those conditions.

### 2.5. Detection of LacZ, sfGFP, and Their Hmp Fusions by SDS-PAGE

The preparation of cells was described earlier in Section 2.3 and Section 2.4. After IPTG induction (3 h for sfGFP, Hmp100-sfGFP, and Hmp-sfGFP, 1 h for LacZ and Hmp-LacZ), cell cultures were centrifuged at 15,000 rpm for 3 min, and resuspended in Milli-Q water. The OD_600_ of each sample was measured to make sure similar cell numbers were loaded into each well. Afterward, samples were mixed with 2× Laemmli Sample Buffer, boiled for 15 min, and centrifuged at 15,000 rpm for 10 min. Supernatants were loaded into the wells of Mini-PROTEAN TGX stain-free precast gels (Bio-Rad Laboratories, Inc., Richmond, CA, USA) for SDS-PAGE, following the manufacturer’s instructions. Protein gels were visualized on a Bio-Rad GelDoc. Densitometry data were generated using ImageJ [39].

### 2.6. β-Casein Expression and Detection by Western Blot

Wild-type harboring pQE80-*β-csn* and pQE80-*hmp-β-csn* were grown from −80 °C frozen stock in 1 mL LB at 37 °C and 250 rpm overnight. After overnight growth, 200 μL of culture was used to inoculate 20 mL of LB media at 37 °C and 250 rpm until it reached OD_600_ of 0.4–0.6 (exponential phase) when cells were induced with 1 mM IPTG. Four hours after induction, cells were harvested by centrifugation at 4000 rpm for 10 min, resuspended in lysis buffer (12.5 mM Tris pH 6.8, 4% SDS), and frozen at −20 °C. Once all samples were collected, they were boiled for 10 min, sonicated (10 s ON, 20 s OFF, repeat 5 times, 30% amplitude using a QSonica Q500 sonicator), and centrifuged at 13,000 rpm for 10 min. The total protein concentration of the supernatant was determined with Pierce™ BCA Protein Assay Kit (Thermo Fisher Scientific, Waltham, MA, USA) and was used to ensure similar amounts of protein were loaded into wells in the following step. The supernatant was mixed with 2× Laemmli Sample Buffer, boiled for 15 min, and loaded on Mini-PROTEAN TGX stain-free precast gels (Bio-Rad Laboratories, Inc, Richmond, CA, USA) for SDS-PAGE, following the manufacturer’s instructions. For Western blots, transfers were done in SDS/Glycine/Methanol buffer for 60 min under 100 V. After the transfers, membranes were blocked with 5% BSA solution for 1 h, and incubated with 6×His Tag Monoclonal Antibody (Invitrogen MA1-21315) (1:2000 dilution) for 1 h. After the membrane was washed with TBST for 5 min five times, the membrane was incubated with the Goat anti-Mouse IgG (H + L) cross-absorbed secondary antibody HRP (Invitrogen 31432) (1:10,000 dilution) for 1 h, and washed again with TBST for 15 min, followed by 5 min washes with TBST for three more times. SuperSignal West Pico PLUS Chemiluminescent Substrate (Thermo Fisher Scientific, Waltham, MA, USA) was used before visualization on a Bio-Rad ChemiDoc. Densitometry data were generated using ImageJ [39].

### 2.7. Protein Degradation Assay

Cells were prepared the same way as for the fluorophore expression assay (Section 2.3). After induction by 1 mM IPTG for 1 h, cells were centrifuged at 15,000 rpm for 3 min, washed with fresh M9 media, diluted to OD_600_ of 0.1, and treated with spectinomycin (100 µg/mL) to prevent further protein synthesis. Cultures were then shaken in a black 96-well plate (Corning Incorporated Inc.) with lid at 37 °C and 250 rpm, and OD_600_ and fluorescence (485/515 nm excitation/emission for sfGFP) were monitored using a SynergyTM H1 Hybrid Microplate Reader.

### 2.8. qPCR

The protocol used for qPCR has been described in detail previously [28,29,40]. Briefly, 30 µL of cell culture (OD_600_ = 0.2) were collected, treated with RNAprotect bacterial reagent (Qiagen, Germantown, MD, USA), centrifuged, and the resulting pellet was frozen at −80 °C. Once all samples were collected, RNA was extracted with RNeasy Mini kits (Qiagen, Germantown, MD, USA) according to instructions from the manufacturer. As an internal standard, 50 ng of purified *phzM* mRNA was added to each sample before extraction. *phzM* mRNA was generated by transcription with a T7 high yield transcription kit (Thermo Fisher Scientific, Waltham, MA, USA) from a linearized pET11a plasmid containing *phzM* from *P. aeruginosa* PAO1. Extracted RNA was converted to cDNA with Taqman reverse transcription kits (Thermo Fisher Scientific, Waltham, MA, USA). Real-time qPCR was performed in a 0.1 mL MicroAMP fast optical 96-well reaction plate with SYBR green and appropriate primers (Table S2) in a ViiA 7 real-time PCR system for 40 cycles (Thermo Fisher Scientific, Waltham, MA, USA). The cycle threshold (Ct) value was used to calculate mRNA abundances in each sample. Calibration curves with pQE80-*gfp_sf_* for *gfp_sf_* and linearized pET11a-*phzM* for *phzM* were used to convert signals to DNA concentrations. *gfp_sf_* cDNA was normalized by *phzM* for comparison between samples.

### 2.9. Protein Solubility Assay

Wild-type harboring pQE80-*β-csn* and pQE80-*hmp-β-csn* were prepared as described in Section 2.6. Wild-type harboring pQE80-*gfp_sf_*, pQE80-*hmp_100_-gfp_sf_*, and pQE80-*hmp-gfp_sf_* were prepared as described in Section 2.3. After 4 h of induction, cell cultures were centrifuged at 4 °C and 4000 rpm for 10 min. Pellets were resuspended in 1 mL of water with 1 mg/mL lysozyme, incubated at 4 °C for 30 min, sonicated on ice (10 s ON, 20 s OFF, repeated 12 times, 30% amplitude using a QSonica Q500 sonicator), and centrifuged again at 4 °C and 8000 rpm for 10 min. The pellets were separated from the supernatant, and 0.5 mL of Milli-Q water was used to resuspend the pellet. Total protein concentrations of samples were determined with Pierce™ BCA Protein Assay Kit (Thermo Fisher Scientific, Waltham, MA, USA), and similar amounts of protein from each sample (for the samples of insoluble fractions, the loaded volume was the same as the total lysate samples and not adjusted based on total protein) were mixed with 2× Laemmli Sample Buffer, boiled for 15 min, and loaded into Mini-PROTEAN TGX stain-free precast gels (Bio-Rad Laboratories, Inc., Richmond, CA, USA) for SDS-PAGE, following the manufacturer’s instructions. Protein gels were visualized on a Bio-Rad GelDoc.

### 2.10. Fusion Tag Cleavage Assay

Protein samples (125 μg) were mixed with 5 µL of enterokinase (New England Biolabs, Ipswich, MA, USA) in 100 µL of reaction buffer (20 mM Tris-HCl, 50 mM NaCl, 2 mM CaCl_2_, pH 8.0) and left at room temperature for 16 h, as instructed by the manufacturer. Samples were then mixed with 2x Laemmli Sample Buffer, boiled for 15 min, and loaded into Mini-PROTEAN TGX stain-free precast gels (Bio-Rad Laboratories, Inc., Richmond, CA, USA) for SDS-PAGE, following the manufacturer’s instructions. Protein gels were visualized on a Bio-Rad GelDoc.

### 2.11. RBS Calculator

The RBS Calculator v2.1 (https://salislab.net/software/predict_rbs_calculator, accessed on 18 November 2021) was used to calculate the translation rate [41]. The mRNA sequences between the transcriptional start site of P_T5_ to the beginning of the lambda t_0_ transcriptional termination sites on pQE80 plasmids were used as the input for mRNA sequence. *E. coli* str. K-12 substr. MG1655 was chosen as the host organism.

## 3. Results

### 3.1. Fusion of Hmp to sfGFP Increases Expression

**Figure 1:**
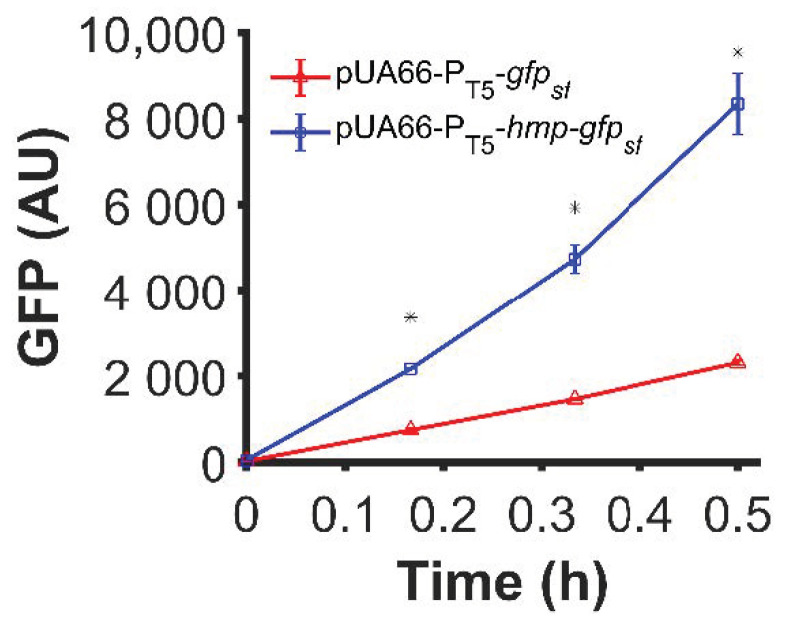
Hmp translational fusion increased expression of sfGFP from an IPTG-inducible promoter under normal growth conditions. Data shown are median GFP signal of wild-type *E. coli* harboring pUA66-P_T5_-*gfp_sf_* (pSA21) (red) and pUA66-P_T5_-*hmp-gfp_sf_* (pJR05) (blue) cultures measured by flow cytometry. Data are the means of three independent replicates, and error bars represent the standard errors of the means. Asterisks (*) indicate statistically significant differences between pUA66-P_T5_-*gfp_sf_* (red) and pUA66-P_T5_-*hmp-gfp_sf_* (blue) as assessed using t-tests and a significance threshold of *p*-value ≤ 0.05.

In previous work, we had observed that translationally fusing Hmp to sfGFP with a (Gly-Ser-Ser-Gly)_3_ linker produced an ~3-fold increase in sfGFP under ·NO stress [28]. Since Hmp is an ·NO-induced stress protein and translation is slower in ·NO-stressed cultures compared to their growing counterparts [42], we tested whether the impact of Hmp on sfGFP expression was generalizable to normal growth conditions. Using a low-copy plasmid (pUA66) where sfGFP and Hmp-sfGFP were expressed from identical IPTG-inducible promoters, we observed a statistically significant 3.6-fold increase in expression for Hmp-sfGFP compared to sfGFP, as measured by fluorescence (Figure 1). These data suggest that the ability of an Hmp translational fusion to increase protein production is translatable to normal, unstressed growth environments.

### 3.2. Fusion to Hmp Increases the Expression of Different Proteins from Different Promoters in Different Plasmids and Different Media

**Figure 2:**
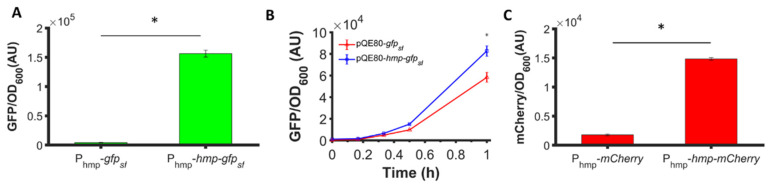
Hmp fusion increases the expression of sfGFP and mCherry. Shown are (**A**) GFP fluorescence measurements normalized by OD_600_ after subtraction of the background signal (wild-type harboring pUA66) at each time point. Depicted are Δ*nsrR* harboring pUA66-P_hmp_-*gfp_sf_* (pXW02) and pUA66-P_hmp_-*hmp-gfp_sf_* (pXW01); (**B**) GFP fluorescence measurements normalized by OD_600_ after subtraction of the background signal (wild-type harboring pQE80L) at each time point. Shown here are wild-type harboring pQE80-P_T5_-*gfp_sf_* (pXW09) and pQE80-P_T5_-*hmp-gfp_sf_* (pXW08) induced by 1 mM IPTG at time 0. Corresponding OD_600_ measurements and data of uninduced samples can be found in Figure S5. (**C**) mCherry fluorescence measurements normalized by OD_600_ after subtraction of the background signal (wild-type harboring pUA66) at each time point. Depicted are Δ*nsrR E. coli* harboring pUA66-P_hmp_-*mCherry* (pXW06) and pUA66-P_hmp_-*hmp-mCherry* (pXW05). Data are the means of three independent replicates, and error bars represent the standard errors of the means. Asterisks (*) indicate statistically significant differences between data as assessed using t-tests and a significance threshold of *p*-value ≤ 0.05.

**Figure 3:**
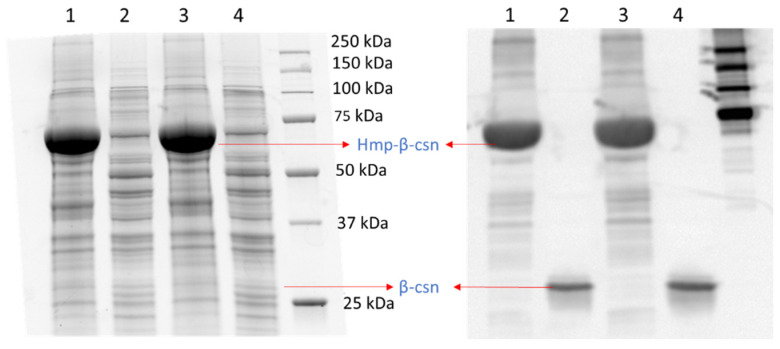
Hmp fusion increases the expression of β-csn. SDS-PAGE (**left**) and Western blot image (**right**) of same protein gel with cell lysates of wild-type *E. coli* harboring pQE80-P_T5_-*hmp-*β*-csn* (pXW12) in lanes 1 and 3, and pQE80-P_T5_-β*-csn* (pXW13) in lanes 2 and 4, with similar total protein quantity after 4 h of induction by 1 mM IPTG. Both Hmp-β-csn and β-csn contained C-terminal 6×His tags and Western blot was conducted with anti-His tag antibodies. Depicted are two independent biological replicates in the same gel.

To assess whether the phenomenon could be observed with different promoters, we used low-copy plasmids where Hmp-sfGFP or sfGFP were expressed from the *hmp* promoter (P_hmp_), and deleted *nsrR* from the chromosome to increase basal expression levels. As depicted in Figure 2A, a significant 37-fold increase in Hmp-sfGFP expression compared to sfGFP expression was observed. To assess whether translational fusion to Hmp could increase expressions from different constructs, we used a different plasmid, which was high-copy (pQE80), and an IPTG-inducible expression cassette. We observed a significant 37% increase in Hmp-sfGFP expression compared to sfGFP (Figure 2B). To confirm that increases in fluorescence corresponded to increases in protein quantity, we ran SDS-PAGE gels on whole cell lysates of cells expressing sfGFP and Hmp-sfGFP, and found the quantity of Hmp-sfGFP to be ~9-fold higher than that of sfGFP, based on densitometry, whereas the size of Hmp-sfGFP was only 3-fold larger than sfGFP (Figure S1). Additional experiments showed that Hmp itself was not fluorescent (Figure S2). To assess whether Hmp fusions to other proteins produced a similar effect, we swapped sfGFP for mCherry in the P_hmp_ expression construct and performed analogous experiments. Notably, a significant 8-fold increase in Hmp-mCherry expression compared to mCherry expression was observed (Figure 2C). To assess the generality of this phenomenon to non-fluorophores and different media, we elected to test whether the fusion of Hmp to β-csn, a cow milk protein that is widely used in the food industry and has potential as a carrier of bioactive agents when it aggregates into micelles [43], could increase expression in LB media. As demonstrated in Figure 3, the expression of Hmp-β-csn was significantly increased by 5.4-fold compared to β-csn by itself, as assessed by Western blot. To assess whether Hmp fusions were amenable to excision by proteases, we had incorporated an enterokinase cleavage site into the linker between Hmp and β-csn (Supplemental Table S3). When Hmp-β-csn was incubated with enterokinase, we found that the fusion protein could be successfully cleaved (Figure S3). These data suggest that Hmp translational fusions can increase the expression of different proteins from different promoters in different plasmids and different media, and that they can be removed from tagged proteins with the incorporation of protease cleavage sites.

### 3.3. Hmp Fusion Proteins Are Largely in the Soluble Fraction of Lysates

**Figure 4:**
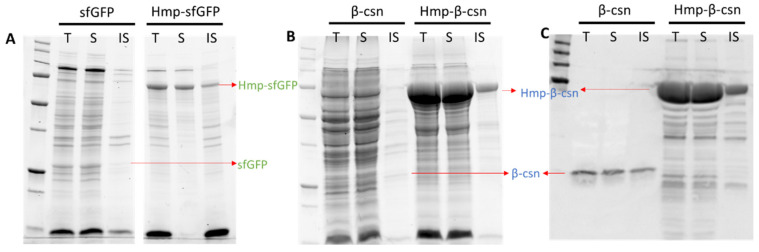
Solubility of sfGFP, Hmp-sfGFP, β-csn, and Hmp-β-csn. After 4 h induction with 1 mM IPTG at 37 °C, wild-type harboring pQE80-P_T5_-*gfp_sf_* (pXW09), pQE80-P_T5_-*hmp-gfp_sf_* (pXW08), pQE80-P_T5_-*β-csn* (pXW13), pQE80-P_T5_-*hmp-β-csn* (pXW12) were sonicated, centrifuged, and separated into soluble (S) and insoluble (IS) fractions. Protein fractions were resolved by SDS-PAGE and visualized through stain-free technique in (**A**,**B**), and Western blot in (**C**). Note that (**B**,**C**) are from the same protein gel. Arrows indicate expected/observed positions of respective protein bands. Both Hmp-β-csn and β-csn contained C-terminal 6×His tags and Western blot was conducted with anti-His tag antibodies. (T) Total cell lysate from induced culture; (S) soluble fraction; (IS) insoluble fraction.

Aggregation into insoluble inclusion bodies can be problematic for over-expressed proteins [44,45]. To determine the solubility status of Hmp fusion proteins, we used centrifugation to separate the soluble and insoluble protein fractions and analyzed them with SDS-PAGE. We found that the majorities of Hmp-sfGFP and Hmp-β-csn were present in the soluble fractions (Figure 4). In consideration that sfGFP and β-csn are largely soluble, these data suggest that translational fusions to Hmp do not appreciably reduce the solubility of expressed proteins. Though we note that whether Hmp fusions increase the solubility of proteins that are prone to aggregation remains to be determined.

### 3.4. Fusion of Hmp to LacZ Does Not Increase Expression

**Figure 5:**
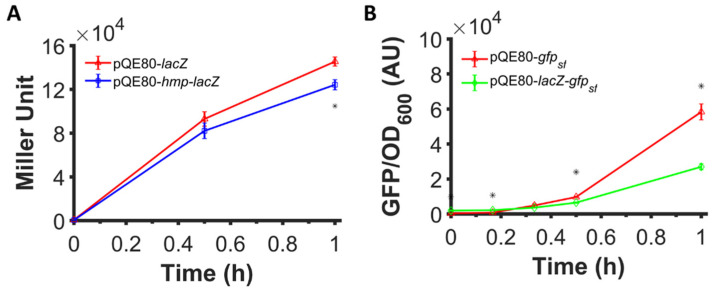
Fusion to Hmp did not boost the expression level of LacZ and fusion to LacZ did not boost the expression of sfGFP. (**A**) Shown are β-galactosidase activities of Δ*lacZYA E. coli* harboring pQE80-P_T5_-*lacZ* (pXW11) and pQE80-P_T5_-*hmp-lacZ* (pXW10) induced with 1 mM IPTG at time 0 after subtraction of the background signal (wild-type harboring pQE80L). (**B**) GFP fluorescence measurements normalized by OD_600_ of wild-type *E. coli* harboring pQE80-P_T5_-*gfp_sf_* (pXW09) and pQE80-P_T5_-*lacZ-gfp_sf_* (pXW22) induced with 1 mM IPTG at time 0 after subtraction of the background signal (wild-type harboring pQE80L). Corresponding OD600 measurements and data of uninduced samples can be found in Figure S5. Data are the means of three independent replicates, and error bars represent the standard errors of the means. Asterisks (*) indicate statistically significant differences between samples as assessed using t-tests and a significance threshold of *p*-value ≤ 0.05.

Figure 2 and Figure 3 illustrate that translational fusion to Hmp increases heterologous protein expression, since sfGFP, mCherry, and β-csn are all foreign to *E. coli*. To assess whether the expression of native *E. coli* proteins can be increased by fusion to Hmp, we assayed the expression of β-galactosidase (LacZ) fused to the C-terminus of Hmp. We expressed LacZ and Hmp-LacZ from an IPTG-inducible promoter on a high copy plasmid (pQE80), and found that the β-galactosidase activity in cells expressing Hmp fused to LacZ was 15% lower than LacZ by itself (Figure 5A). To assess the protein quantity, we ran an SDS-PAGE on whole cell lysates of cells expressing LacZ and Hmp-LacZ, and found their quantity to be comparable using densitometry, with Hmp-LacZ approximately 24% lower than LacZ by itself (Figure S1). These data suggest that the fusion of Hmp to a native *E. coli* protein, LacZ, did not boost its expression. Further, these data raised the question of whether Hmp fusions increased heterologous protein expression in *E. coli* only because it was a native protein, or whether the effect was more specific to Hmp itself. To address this question, we generated a LacZ-sfGFP, where Hmp in the fusion protein was replaced with LacZ, and measured fluorescence after IPTG induction. We observed that the expression of LacZ-sfGFP was ~2-fold lower than sfGFP by itself (Figure 5B). These data suggest that Hmp fusions increase heterologous protein expression through a mechanism that is not shared by all native *E. coli* proteins.

### 3.5. Catalytic Activity Is Not Required to Observe Expression Enhancement from Hmp Fusions

**Figure 6:**
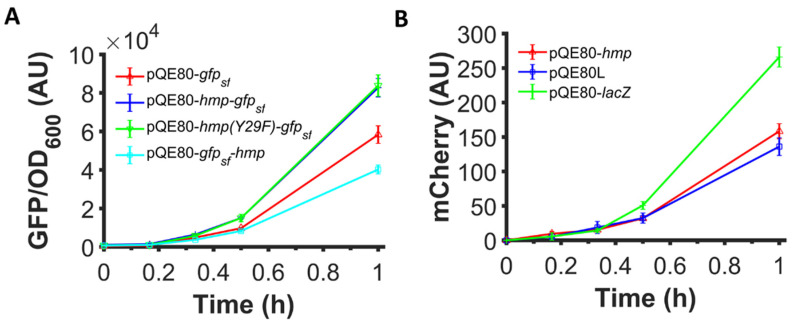
N-terminal fusion is required and O_2_ binding and ·NO detoxification activities are dispensable for Hmp to boost protein expression. Shown are (**A**) GFP fluorescence normalized by OD_600_ after subtraction of the background signal (wild-type harboring pQE80L) of wild-type *E. coli* harboring pQE80-P_T5_-*gfp_sf_* (pXW09)*,* pQE80-P_T5_-*hmp-gfp_sf_* (pXW08)*,* pQE80-P_T5_-*hmp(Y29F)-gfp_sf_* (pXW14), whose O_2_-binding site is destroyed, and pQE80-P_T5_-*gfp_sf_-hmp* (pXW15) induced by 1 mM IPTG at time 0. (**B**) mCherry fluorescence of MO001 (*lacIq*, Δ*lacZYA*::P_T5_*-mCherry*) harboring pQE80-P_T5_-*hmp* (pXW20)*,* pQE80L (empty vector), and pQE80-P_T5_-*lacZ* (pXW11) induced by 1 mM IPTG at time 0. Corresponding OD_600_ measurements and data of uninduced samples can be found in Figure S5. Data are the means of at least three independent replicates, and error bars represent the standard errors of the means.

Given that previous studies found that VHb expression increased growth rate and protein yield through a mechanism that was suggested to involve its O_2_ binding or ·NO detoxification abilities, we tested whether the increased expression observed with Hmp fusions involved those functions. Specifically, we used a catalytically-inactive Hmp, Hmp(Y29F) whose O_2_ dissociation rate is increased by 80-fold and ·NO detoxification activity is reduced by 30-fold, and translationally fused it with sfGFP. The expression of Hmp(Y29F)-sfGFP fusion from an IPTG-inducible promoter was found to be similar to the expression of Hmp-sfGFP (Figure 6A), indicating that the ·NO defense and O_2_-binding capabilities of Hmp were not required to observe its ability to increase heterologous protein expression.

### 3.6. Fusion of Hmp to the N-Terminus of Proteins Is Required for Increased Expression

Previous work that showed that VHb increased growth rate and product yield was done with VHb expressed as an independent protein. To assess whether Hmp can promote the expression of proteins without being fused to them, we used a genomically-integrated, IPTG-inducible mCherry system and expressed Hmp from an IPTG-inducible promoter on a plasmid. We found that the expression of mCherry was not higher when Hmp was expressed compared to an empty vector control or a control that expressed LacZ in place of Hmp from the same plasmid (Figure 6B). To assess whether N- or C-terminal fusions to Hmp could increase protein expression, we constructed a plasmid expressing Hmp translationally fused to the C-terminus of sfGFP, instead of the N-terminus, and found that sfGFP-Hmp production was lower than that of sfGFP (Figure 6A). These data indicate that Hmp only boosts heterologous protein expression when it is translationally fused to the N-terminus of the target protein.

### 3.7. Increased Expression Is Not Due to a Second Start Codon

**Figure 7:**
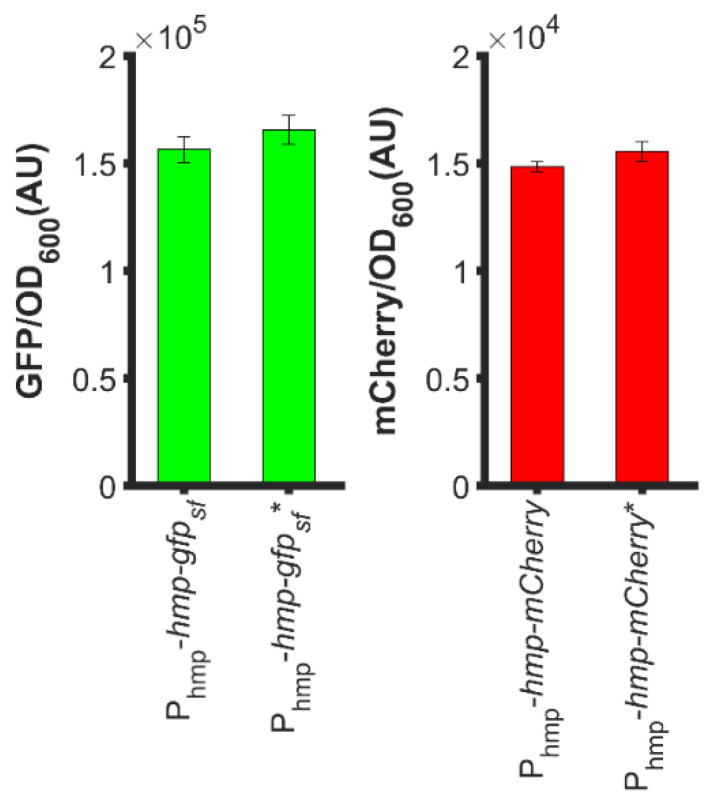
Second start codons of the translational fusions did not impact expression levels appreciably. (**Left**): GFP fluorescence normalized by OD_600_ after subtraction of the background signal (wild-type harboring pUA66) of exponential-phase Δ*nsrR E. coli* harboring pUA66-P_hmp_-*hmp-gfp_sf_* (pXW01) and pUA66-P_hmp_-*hmp-gfp_sf_* * (pXW04, start codon of *gfp_sf_* removed); (**Right**): mCherry fluorescence normalized by OD_600_ after subtraction of the background signal (wild-type harboring pUA66) of exponential-phase Δ*nsrR E. coli* harboring pUA66-P_hmp_-*hmp-mCherry* (pXW05) and pUA66-P_hmp_-*hmp-mCherry* * (pXW07, start codon of *mCherry* removed). Data are the means of three independent replicates, and error bars represent the standard errors of the means.

To understand how N-terminal Hmp fusions increased heterologous protein expression, we considered whether the start codon of target proteins contributed to fluorescence or catalytic activity measurements. With the concern that the second start codons (ATG) of *hmp-gfp_sf_* and *hmp-mCherry* translational fusions, which were situated at the beginning of *gfp_sf_* and *mCherry*, would promote expression, we removed those start codons from the translational fusion plasmids, and found minimal impact on the overall sfGFP or mCherry level (Figure 7). Overall, these data suggest that the increased expression of Hmp fusions cannot be explained by the presence of second start codons.

### 3.8. RBS Calculator Predicts the Impact of an N-Terminal Linker but Not the Increased Expression of Hmp Fusions

To assess whether the linker peptide (Gly-Ser-Ser-Gly)_3_ contributed to increased protein expression in fusion proteins, we removed only Hmp from Hmp-sfGFP, which left sfGFP preceded by the (Gly-Ser-Ser-Gly)_3_ linker. Surprisingly, we found that the linker peptide significantly reduced fluorescence by more than 18-fold (Figure S2). To understand this phenomenon, we calculated the protein expression rate of our constructs with an RBS calculator [41,46], which predicts the translational initiation and protein expression based on a free energy model. As shown in Figure S4, the calculator predicted that when the linker peptide is inserted at the N-terminus of sfGFP, its expression should be reduced by ~70 fold, which qualitatively agrees with the trend we observed. However, the RBS calculator also predicted that the expression of sfGFP should be ~5 fold higher than the expression of Hmp-sfGFP, which was not the case. These data suggest that the expression enhancement of Hmp fusion proteins was not due to the linker peptide, and it is unlikely to be explained by differences in translation initiation.

### 3.9. Transcript Levels and Protein Degradation Are Comparable between Hmp-sfGFP and sfGFP

**Figure 8:**
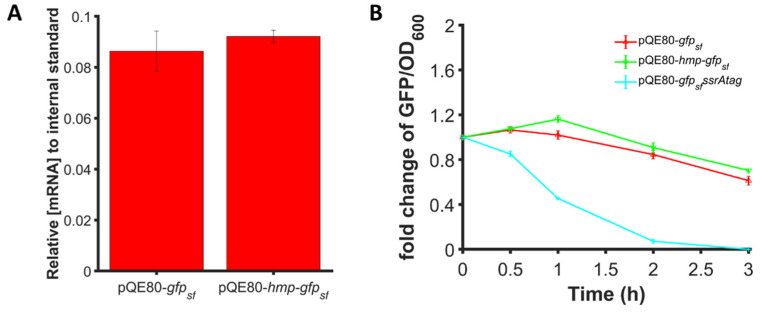
Expression enhancement is not due to transcript or protein degradation differences. (**A**) Transcript levels of *gfp_sf_* in wild-type harboring pQE80-P_T5_-*gfp_sf_* (pXW09)*,* pQE80-P_T5_-*hmp-gfp_sf_* (pXW08) after 1 h induction of 1 mM IPTG. Transcript levels shown are relative to the *phzM* internal standard spiked into each sample before RNA extraction. (**B**) Fold change of GFP/OD_600_ after subtraction of the background signal (wild-type harboring pQE80L) of wild-type harboring pQE80-P_T5_-*gfp_sf_* (pXW09)*,* pQE80-P_T5_-*hmp-gfp_sf_* (pXW08)*,* and pQE80-P_T5_*-gfp_sf_ssrAtag* (pXW21) following treatment with 100 µg/mL spectinomycin to stop translation. Cells were induced with 1 mM IPTG for 1 h before spectinomycin treatment. Data are the means of at least three independent replicates, and error bars represent the standard errors of the means.

We considered whether differences in transcript levels or protein degradation could explain the enhancements in protein expression observed for Hmp fusion proteins. As shown in Figure 8A, we found that 1 mM IPTG induction generated similar *gfp_sf_* transcript levels when expressed from P_T5_-*hmp-gfp_sf_* or P_T5_-*gfp_sf_* even though the protein levels were found to be 37% higher for P_T5_-*hmp-gfp_sf_* (Figure 2B). Using spectinomycin to halt translation, we found that the degradation of Hmp-sfGFP and sfGFP were also similar (Figure 8B). Collectively, these results indicated that the increased expression of Hmp fusions was not attributable to differences in transcript levels or protein degradation, and thus suggested that Hmp-sfGFP was translated more efficiently per transcript compared to sfGFP.

### 3.10. Fusions of Truncated Versions of Hmp Also Increase Expression

**Figure 9:**
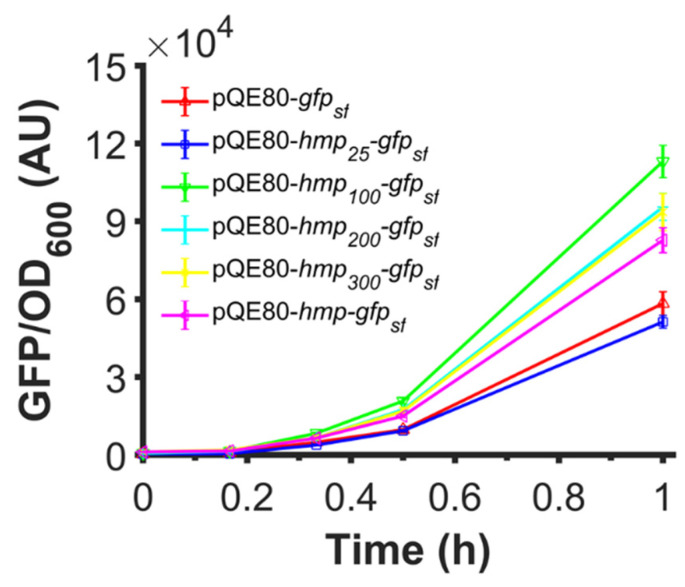
Fusions of truncated versions of Hmp that are as long as 100 AA also increase expression. Shown are GFP fluorescence normalized by OD_600_ after subtraction of the background signal (wild-type harboring pQE80L) of wild-type harboring pQE80-P_T5_-*gfp_sf_* (pXW09)*,* pQE80-P_T5_-*hmp_25_-gfp_sf_* (pXW16)*,* pQE80-P_T5_-*hmp_100_-gfp_sf_* (pXW17)*,* pQE80-P_T5_-*hmp_200_-gfp_sf_* (pXW18)*,* pQE80-P_T5_-*hmp_300_-gfp_sf_* (pXW19)*,* pQE80-P_T5_-*hmp-gfp_sf_* (pXW08) after induction of 1 mM IPTG at time 0. Corresponding OD_600_ measurements and data of uninduced samples can be found in Figure S5. Data are the means of at least three independent replicates, and error bars represent the standard errors of the means.

Since the catalytic activity of Hmp was not required to observe an increase in expression, and data suggested that translation efficiency was responsible for the observed effects, we investigated whether truncated versions of Hmp could increase protein expression when fused to the N-terminus of proteins. To accomplish this, we used plasmids where different truncations of Hmp (e.g., Hmp_25_ is the first 25 amino acids of Hmp) were fused to the N-terminus of sfGFP using a (Gly-Ser-Ser-Gly)_3_ linker. We observed that while Hmp_25_-sfGFP reduced the protein expression compared to sfGFP alone, all other truncations improved the expression compared to sfGFP and Hmp-sfGFP (Figure 9). Notably, Hmp_100_-sfGFP was significantly higher than all other constructs.

## 4. Discussion

Recombinant protein constitutes a large global market, with more than 100 billion dollars in sales of therapeutic proteins and more than 5 billion dollars in industrial enzymes [47,48,49]. Protein fusion tags are essential tools in recombinant protein expression to improve the yield and solubility of heterologous proteins, enable the purification of target proteins, and specify the location of protein expression within the cell [50]. Many fusion tags, including MBP [51,52], NusA [53], and Trx [54] from *E. coli*; CpcB [55,56] from *Synechocystis*; SUMO [57] from *Homo sapiens*; and GST [58] from *Schistosoma japonicum*, are found to increase expression yield when fused to the N-terminus [59]. Here, we identified Hmp from *E. coli* as an N-terminal fusion tag that can increase protein expression in *E. coli*. Importantly, *E. coli* is one of the most widely used recombinant protein expression systems, due to its lower production cost and genetic tractability [60]. We found that N-terminal Hmp fusions increase the expression of different proteins, from different plasmids, with different promoters, and in different media (Figure 1, Figure 2 and Figure 3). However, this effect was not present for fusions to *lacZ*, which is native to *E. coli* (Figure 5A). While the lack of enhancement in LacZ expression was not further investigated here, we speculate that its role as a native, core metabolic enzyme for growth on lactose may have honed its translational efficiency to approach near-optimal levels, whereas heterologous proteins have not been subjected to the same evolutionary pressures for expression in *E. coli*. However, it is important to note that additional proteins native to *E. coli* will have to be tested with Hmp fusions to their N-terminus to determine if LacZ is an outlier or representative *E. coli* protein in this regard.

To investigate the mechanism of this phenomenon, we found that the catalytic activity and O_2_-binding abilities of Hmp were not required, and that Hmp had to be fused to the N-terminus of the protein rather than as a C-terminal fusion or separately expressed protein (Figure 6). Although, we note that the experiments performed here were aerobic and whether this phenomenon occurs in anaerobic environments has yet to be determined. The results presented here are in contrast to the application of VHb, where it is expressed as an independent protein (not as a fusion) to improve the overall health of the cell, and its O_2_-binding properties or nitrosative stress defenses have been proposed to play a role in its ability to boost growth and protein production [21,22,24,27]. In addition, experiments with truncations of Hmp suggested that the portion of Hmp required to observe the phenomenon (Hmp_100_) was devoid of the FAD- and NAD(P)-binding sites, and thus unable to detoxify ·NO [61]. Alternatively, we found that transcript levels and protein degradation were not involved in the enhancement in protein abundance due to fusion with Hmp, but rather that it is likely due to an increase in translation rate (Figure 8). Further, the predicted translation rate with the RBS calculator (Figure S4) suggested that translation initiation is unlikely to be the translational step responsible for the enhancement.

Stress response proteins, including RpoA [62], SlyD [63], Tsf [64], RpoS [65], PotD [66] and Crr [66], have been suggested to be used as solubility enhancers, as their native function would require them to be properly folded under stressful conditions, which often lead to protein aggregation and misfolding. Here we found that the majority of Hmp-GFP and Hmp-β-csn expressed were soluble, which was expected because both sfGFP and β-csn are fairly soluble by themselves (Figure 4) [67,68]. The extent to which N-terminal Hmp fusions can enhance the expression of insoluble proteins has yet to be assessed and represents a fertile area of future study. Collectively, the data presented here suggest that Hmp, a nitrosative stress response protein, or its first 100 amino acids, have the potential to increase the expression of heterologous proteins when used as an N-terminal fusion tag. Whether this is also true for other microbial hemoglobins, or whether this can be applied to *P. pastoris* or other eukaryotic cells has yet to be determined; however, these data suggest that there is an unappreciated dimension of how bacterial hemoglobins can be deployed as biotechnological tools.

## Data Availability

The data presented in this study are available in the main text and supplemental material.

## Supplementary Materials

The following are available online at https://www.mdpi.com/article/10.3390/microorganisms10020358/s1, Figure S1: Visualization of LacZ, Hmp-LacZ, sfGFP, Hmp_100_-sfGFP, and Hmp-sfGFP with SDS-PAGE of whole cell lysates.; Figure S2: Expression of GFP preceded by the linker peptide is low and Hmp is not fluorescent; Figure S3: Hmp-β-csn with an enterokinase cleavage site cleaved by enterokinase to yield β-csn; Figure S4: Predicted translation rate of constructs used in this study by RBS calculator v2.1; Figure S5: Fluorescence measurements of uninduced controls and the OD_600_ measurement of induced samples; Table S1: Bacterial strain and plasmid table; Table S2: DNA primer sequence. Table S3: Amino acid sequences of proteins used in this study.

## Abbreviations

Hmp: *E. coli* flavohemoprotein; β-csn: bovine β-casein; LacZ: *E. coli* β-galactosidase; ·NO: nitric oxide; VHb: *Vitreoscilla* hemoglobin; sfGFP: superfold green fluorescent protein; IPTG: isopropropyl-β-D-thiogalactopyranoside; MBP: *E. coli* maltose-binding protein; GST: glutathione S-transferase; TRX: thioredoxin; RBS: ribosomal binding site; qPCR: quantitative polymerase chain reaction; SDS-PAGE: sodium dodecyl sulfate-polyacrylamide gel electrophoresis; HRP: horseradish peroxidase; FAD: flavin adenine dinucleotide; NAD: nicotinamide adenine dinucleotide; MU: Miller units; OD: optical density.
