# Supplementary material for: Translational Fusion to Hmp Improves Heterologous Protein Expression
Source: Microorganisms. 2022 Feb 4;10(2):358. doi: 10.3390/microorganisms10020358 (PMC8879370; doi:10.3390/microorganisms10020358)
Supplement: Supplementary file 1 [file microorganisms-10-00358-s001.zip › microorganisms-1532406-supplementary.pdf]

Table S1. Bacterial strain and plasmid table. Bacterial strains and plasmids used in this study.

**Strain**	**Genotype**	**Source**
MG1655	F- λ- *ilvG*- *rfb*-50 *rph*-1	ATCC 700926 [29]
XW011	MG1655 Δ*nsrR*	This work[30][a, c]
XW012	MG1655 Δ*lacZYA*	This work[b, c]
MO001	MG1655 *lacI::lacI*^{q} Δ*lacZYA::*P_{T5}-*mcherry*	[31]
**Plasmid**	**Genotype**	**Source**
pQE80L	Vector, ColE1*ori*, P_{T5} (IPTG-inducible), *lacI*^{q}, a*mp*^{R}	Qiagen, Germantown, MD, United States
pUA66	Vector, Sc101*ori*, *gfp*mut2(promoterless), *kan*^{R}	[32]
pET11-a-*phzM*	pET11-a P_{T7}-*phzM*	[33]
pJR05	pUA66 P_{T5}-*hmp-(GSSG)*$_3$-*gfp*$_{sf}$, *lacI*^{q}	[29]
pSA21	pUA66 P_{T5}-*gfp*$_{sf}$, *lacI*^{q}	[34]
pXW01	pUA66 P_{hmp}-*hmp-(GSSG)*$_3$-*gfp*$_{sf}$	[28]
pXW02	pUA66 P_{hmp}-*gfp*$_{sf}$	[28]
pXW04	pUA66 P_{hmp}-*hmp-(GSSG)*$_3$-*gfp*$_{sf}$* [d]	This work[e]
pXW05	pUA66 P_{hmp}-*hmp-(GSSG)*$_3$-*mcherry*	This work[f]
pXW06	pUA66 P_{hmp}-*mcherry*	This work[e]
pXW07	pUA66 P_{hmp}-*hmp-(GSSG)*$_3$-*mcherry** [d]	This work[e]
pXW08	pQE80 P_{T5}-*hmp-(GSSG)*$_3$-*gfp*$_{sf}$, *lacI*^{q} [d]	This work[f]
pXW09	pQE80 P_{T5}-*gfp*$_{sf}$, *lacI*^{q}	This work[e]
pXW10	pQE80 P_{T5}-*hmp-(GSSG)*$_3$-*lacZ-6xHis, lacI*^{q} [d]	This work[f]
pXW11	pQE80 P_{T5}-*lacZ-6xHis lacI*^{q}	This work[e]
pXW12	pQE80 P_{T5}-*hmp-(GSSG)*$_3$-*(DDDDK)-β-csn-6xHis lacI*^{q} [d, g]	This work[f]
pXW13	pQE80 P_{T5}-*β-csn-6xHis lacI*^{q}	This work[e]
pXW14	pQE80 P_{T5}-*hmp(Y29F)- (GSSG)*$_3$-*gfp*$_{sf}$, *lacI*^{q} [d]	This work[e]
pXW15	pQE80 P_{T5}-*gfp*$_{sf}$-*(GSSG)*$_3$-*hmp*, *lacI*^{q} [d]	This work[f]
pXW16	pQE80 P_{T5}-*hmp(1-25)- (GSSG)*$_3$-*gfp*$_{sf}$, *lacI*^{q} [d]	This work[e]
pXW17	pQE80 P_{T5}-*hmp(1-100)- (GSSG)*$_3$-*gfp*$_{sf}$, *lacI*^{q} [d]	This work[e]
pXW18	pQE80 P_{T5}-*hmp(1-200)- (GSSG)*$_3$-*gfp*$_{sf}$, *lacI*^{q} [d]	This work[e]
pXW19	pQE80 P_{T5}-*hmp(1-300)- (GSSG)*$_3$-*gfp*$_{sf}$, *lacI*^{q} [d]	This work[e]

pXW20	pQE80 P_{T5}-*hmp*, *lacI*^{q}	This work[e]
pXW21	pQE80 P_{T5}-*gfp*$_{sf}$-*ssrA*$_{tag}$, *lacI*^{q}	This work[e]
pXW22	pQE80 P_{T5}-*lacZ-(GSSG)*$_{3}$-*gfp*$_{sf}$, *lacI*^{q}	This work[f]
pXW23	pQE80 P_{T5}-*M-(GSSG)*$_{3}$-*gfp*$_{sf}$	This work[e]

a. generated with P1 transduction from the Keio collection [30] or previously constructed strain to strain indicated.
b. generated with the λ Red system (pKD46)[35] with the primers specified in Table S2.
c. *kan*^{R} was removed (cured) using pCP20[36].
d. start codon (ATG) of the protein whose N-terminus is fused to the C-terminus of another protein was removed. For β-casein, the signal peptide at N terminus, which has the start codon of the protein, was not included.
e. constructed with primers described in Table S2 and Q5 SDM kit (NEB).
f. constructed with primers described in Table S2 and Hifi DNA assembly kit (NEB).
g. includes an enterokinase cleavage site.

Table S2. DNA primer sequence. Primers used in this study to, A) construct mutants, B) confirm deletions, C) construct plasmids, and D) perform qPCR.

A. Mutant Construction

Mutation	Forward Primer	Reverse Primer
MG1655 Δ*lacZYA*	GGAATTGTGAGCGGATA ACAATTTCACACAGGAA ACAGCTGAGCGATTGTG TAGGCTGGA	TAGGCCTGATAAGCGCA GCGTATCAGGCAATTTTT ATAATTTAACGGCTGAC ATGGGAAT

Primers consisted of a 40 nt homology region upstream and downstream of lac operon in chromosome, and ~20 nt sequence homologous to *kanR* cassette on pKD13[35].

B. Mutant Verification

Mutation	Upstream Forward Primer	Downstream Reverse Primer	Internal Forward Primer	Internal Reverse Primer
Δ*nsrR*	TTTTTCCTTCC CCGAACTGA	ATATTGTCGC CCAGCACTTC	GGATTACGTG CGCTGATCTA	AAAGCGGTTG ATTCTCTTCAA
Δ*lacZYA*	CGCAAACCGC CTCTCCCCGCG	GCGTAGTATC AGCGGCAATT	AACGTCGTGA CTGGGAAAAC	GAAATAATAG TGCTTATCCCG GTC
kanR				GAAGCGGTCA GCCCATTC

kanR internal reverse primer was used with upstream forward primer to confirm gene deletion and resistant marker insertion.

C. Plasmid Construction

Plasmid	Genotype	Forward primer	Reverse Primer	Description
pXW04	pUA66 P_{hmp}-*hmp*-*(GSSG)*$_3$-*gfp*$_{sf}$*	AGCAAAGGA GAAGAACTTT TCACTGG	CCCACTGCT CCCACCAGA	Remove the ATG on pXW01 using Q5 SDM kit (NEB)
pXW05 (vector)	pUA66 P_{hmp}-*hmp*-*(GSSG)*$_3$-*mcherry*	GCTGTACAAG TAACCTGCAG GCATGCAAGC	TGCTCACCA TCCCACTGC TCCCACC	Amplify the pUA66-P_{hmp}-*hmp* vector from pXW01 with Phusion to be used in Hifi DNA assembly reaction.
pXW05 (insertion)		GAGCAGTGG GATGGTGAGC AAGGGCGAG	CTGCAGGTT ACTTGTACA GCTCGTCCA TGCC	Amplify the *mcherry* insertion from MO001 genome with Phusion to be used in Hifi DNA assembly reaction.
pXW06 (vector)	pUA66 P_{hmp}-*mcherry*	GCTGTACAAG TAACCTGCAG GCATGCAAGC	TGCTCACCA TATGGTCTT CCTTTTTTT GCATCTTAA TTGATG	Amplify the pUA66-P_{hmp} vector from pXW02 with Phusion to be used in Hifi DNA assembly reaction.

pXW06 (insertion)		GGAAGACCATATGGTGAGCAAGGGCGAG	CTGCAGGTTACTTGTACAGCTCGTCCATGCC	Amplify the *mcherry* insertion from MO001 genome with Phusion to be used in Hifi DNA assembly reaction.
pXW07	pUA66 P_{hmp}-*hmp-(GSSG)*$_3$-*mcherry**	AGCAAAGGAGAAGAACTTTTCACTGG	CCCACTGCTCCCACCAGA	Remove the ATG on pXW05 using Q5 SDM kit (NEB)
pXW08 (vector)	pQE80 P_{T5}-*hmp-(GSSG)*$_3$-*gfp*$_{sf}$, *lacI*^{q}	CTACAAATAACTGAGCTTGGACTCCTGTTGATAGA	GCGTCAAGCATAGTTAATTTCTCCTCTTTAATGAATTCTGTGTGAAA	Amplify the pQE80-P_{T5} from pQE80L with Phusion to be used in Hifi DNA assembly reaction.
pXW08 (insertion)		GAAATTAACTATGCTTGACGCTCAAACCATCG	TCCAAGCTCAGTTATTTGTAGAGCTCATCCATGCCAT	Amplify the *hmp-(GSSG)*$_3$-*gfp*$_{sf}$ insertion from pXW04 with Phusion to be used in Hifi DNA assembly reaction.
pXW09	pQE80 P_{T5}-*gfp*$_{sf}$, *lacI*^{q}	AGCAAAGGAGAAGAACTTTTCACTGG	CATAGTTAATTTCTCCTCTTTAATGAATTCTGTGTG	Remove the *hmp-(GSSG)*$_3$ on pXW08 using Q5 SDM kit (NEB)
pXW10 (vector)	pQE80 P_{T5}-*hmp-(GSSG)*$_3$-*lacZ lacI*^{q}	CACCATCACCATCACCATTAACTGAGCTTGGACTCCTGTTG	TAATCATGGTCCCACTGCTCCCACC	Amplify the pQE80-P_{T5}-*hmp-(GSSG)*$_3$ from pXW08 with Phusion to be used in Hifi DNA assembly reaction.
pXW10 (insertion)		GAGCAGTGGGACCATGATTACGGATTCACTGG	AATGGTGATGGTGATGGTGTTTTTGACACCAGACCAACTGGTAATGGT	Amplify the *lacZ* insertion from WT genome with Phusion to be used in Hifi DNA assembly reaction.
pXW11	pQE80 P_{T5}-*lacZ lacI*^{q}	ACCATGATTACGGATTCACTGGCC	CATAGTTAATTTCTCCTCTTTAATGAATTCTGTGTG	Remove the *hmp-(GSSG)*$_3$ on pXW10 using Q5 SDM kit (NEB)
pXW12 (vector)	pQE80 P_{T5}-*hmp-(GSSG)*$_3$-*β-csn lacI*^{q}	TCACCATCACTAGCTGAGCTTGGACTCCTGTTGA	GAGCGTCAAGCATAGTTAATTTCTCCTCTTTAATGAATTCTGTGTGAAA	Amplify the pQE80-P_{T5} from pQE80L with Phusion to be used in Hifi DNA assembly reaction.

pXW12 (insertion 1)		ATTAACTATGCTTGACGCTCAAACCATCG	CTAATTCCCTCTTGTCATCGTCATCCCCACTGCTCCCACCAG	Amplify the *hmp-(GSSG)*$_3$*-(DDDDK)* insertion from pXW08 with Phusion to be used in Hifi DNA assembly reaction.
pXW12 (insertion 2)		CGATGACAAGAGGGAATTAGAAGAGTTGAACGTCC	AGCTCAGCTAGTGATGGTGATGGTGATGCACGATGATTGGGAATGGACC	Amplify the *β-csn* insertion from synthetic DNA (Genewiz) with Phusion to be used in Hifi DNA assembly reaction.
pXW13 (vector)	pQE80 P_{T5}-*β-csn* *lacI*^{q}	TCACCATCACTAGCTGAGCTTGGACTCCTGTTGA	CTTCTAATTCCCTCATAGTTAATTTCTCCTCTTTAATGAATTCTGTGTG	Amplify the pQE80-P_{T5} vector from pQE80L with Phusion to be used in Hifi DNA assembly reaction.
pXW13 (insertion)		GAAATTAACTATGAGGGAATTAGAAGAGTTGAACGTCC	AGCTCAGCTAGTGATGGTGATGGTGATGCACGATGATTGGGAATGGACC	Amplify the *β-csn* insertion from synthetic DNA (Genewiz) with Phusion to be used in Hifi DNA assembly reaction.
pXW14	pQE80 P_{T5}-*hmp(Y29F)-(GSSG)*$_3$-*gfp*$_{sf}$, *lacI*^{q}	GCCCATTTCTTCGACCGTATG	GGTTAACTTTGGCCCCGT	Mutate the 29th codon of *hmp* open reading frame in pXW08 from TAC to TTC using Q5 Site-directed Mutagenesis kit (Qiagen).
pXW15 (vector)	pQE80 P_{T5}-*gfp*$_{sf}$-*(GSSG)*$_3$-*hmp*, *lacI*^{q}	TAAGGTGCTGTAACTGAGCTTGGACTCCTGTTGA	TCTCCTTTGCTCATAGTTAATTTCTCCTCTTTAATGAATTCTGTGT	Amplify the pQE80-P_{T5} from pQE80L with Phusion to be used in Hifi DNA assembly reaction.
pXW15 (insertion 1)		AATTAACTATGAGCAAAGGAGAAGAACTTTTCACTGG	CCACCAGAACTACCGCCGGAAGAGCCTTTGTAGAGCTCATCCATGCCATG	Amplify the *gfp*$_{sf}$ insertion from pXW08 with Phusion to be used in Hifi DNA assembly reaction.
pXW15 (insertion 2)		TCCGGCGGTAGTTCTGGTGGGAGCAGTGGGCTTGACGCT	CCAAGCTCAGTTACAGCACCTTATGCGGGC	Amplify the *hmp* insertion from pXW08 with Phusion to be used in Hifi DNA assembly reaction.

		CAAACCATCGC		
pXW16	pQE80 P_{T5}-*hmp(1-25)-(GSSG)$_3$-gfp$_{sf}$*, *lacI^q*	GGCTCTTCCGGCGGTAGTTCTGGTG	GGTTAACTTTGGCCCCGTTTCCACCAGTAA	Remove the 26-396th amino acids of *hmp* on pXW08 using Q5 SDM kit (NEB)
pXW17	pQE80 P_{T5}-*hmp(1-100)-(GSSG)$_3$-gfp$_{sf}$*, *lacI^q*	GGCTCTTCCGGCGGTAGTTCTGGTG	TTCACCGACGATGTTGTACTGTTCC	Remove the 101-396th amino acids of *hmp* on pXW08 using Q5 SDM kit (NEB)
pXW18	pQE80 P_{T5}-*hmp(1-200)-(GSSG)$_3$-gfp$_{sf}$*, *lacI^q*	GGCTCTTCCGGCGGTAGTTCTGGTG	ATGTGGGAAACCTTCCGGCTTCAGCCAGAC	Remove the 201-396th amino acids of *hmp* on pXW08 using Q5 SDM kit (NEB)
pXW19	pQE80 P_{T5}-*hmp(1-300)-(GSSG)$_3$-gfp$_{sf}$*, *lacI^q*	GGCTCTTCCGGCGGTAGTTCTGGTG	ATCGCCATTTTCTGCCGCATGGAAC	Remove the 301-396th amino acids of *hmp* on pXW08 using Q5 SDM kit (NEB)
pXW20	pQE80 P_{T5}-*hmp*, *lacI^q*	CAGCACCTTATGCGGGCCAAAG	TAACTGAGCTTGGACTCCTGTTGATAGA	Remove *gfp$_{sf}$* from pXW08 using Q5 SDM kit (NEB)
pXW21	pQE80 P_{T5}-*gfp$_{sf}$-ssrAtag*, *lacI^q*	CTACGCTTTAGCAGCTTAACTGAGCTTGGACTCC	CTACGCTTTAGCAGCTTAACTGAGCTTGGACTCC	Insert *ssrA* tag (Ala Ala Asn Asp Glu Asn Tyr Ala Leu Ala Ala) on the end of *gfp$_{sf}$* using Q5 SDM kit (NEB)
pXW22 (vector)	pQE80 P_{T5}-*lacZ-(GSSG)$_3$-gfp$_{sf}$*, *lacI^q*	GCTCTACAAATAACTGAGCTTGGACTCCTGTTGA	CGGAAGAGCCTTTTTGACACCAGACCAACTGGTAATG	Amplify the (GSSG)$_3$-*gfp$_{sf}$* insertion from pXW08 with Phusion to be used in Hifi DNA assembly reaction.
pXW22 (insertion)		GTGTCAAAAAGGCTCTTCCGGCGGTAG	AGCTCAGTTATTTGTAGAGCTCATCCA	Amplify the pQE80 P_{T5}-*lacZ*-vector from pXW11 with

				Phusion to be used in Hifi DNA assembly reaction.
pXW23	pQE80 P_{T5}-*M-(GSSG)$_3$-gfp$_{sf}$*	GGCTCTTCCG GCGGTAGTTC TGGTG	CATAGTTAA TTTCTCCTC TTTAATGAA TTCTGTGTG AAATTGT	Remove *hmp* from pXW08 using Q5 SDM kit (NEB)

D. qPCR primers

Target gene	Forward Primer	Reverse Primer
phzM	CGGCGAAGACTTCTACA GCTA	CAGGATGGCCTTGGTCA AT
gfp$_{sf}$	GGTGATGTTAATGGGCA CAA	GGCCACGGAACAGGTAG TTT

Table S3. Amino acid sequences of proteins used in this study.

Protein Name	Plasmid Name	Amino acid sequence
sfGFP	pSA21, pXW02, pXW09	MSKGEELFTGVVPILVELDGDVNGHKFSVRGEGEGDATNGK LTLKFICTTGKLPVPWPTLVTTLTYGVQCFSRYPDHMKRHD FFKSAMPEGYVQERTISFKDDGTYKTRAEVKFEGDTLVNRIE LKGIDFKEDGNILGHKLEYNFNSHNVYITADKQKNGIKANF KIRHNVEDGSVQLADHYQQNTPIGDGPVLLPDNHYLSTQSV LSKDPNEKRDHMVLLEFVTAAGITHGMDELYK
sfGFP-ssrAtag	pXW21	MSKGEELFTGVVPILVELDGDVNGHKFSVRGEGEGDATNGK LTLKFICTTGKLPVPWPTLVTTLTYGVQCFSRYPDHMKRHD FFKSAMPEGYVQERTISFKDDGTYKTRAEVKFEGDTLVNRIE LKGIDFKEDGNILGHKLEYNFNSHNVYITADKQKNGIKANF KIRHNVEDGSVQLADHYQQNTPIGDGPVLLPDNHYLSTQSV LSKDPNEKRDHMVLLEFVTAAGITHGMDELYKAANDENYA LAA
M-Linker-sfGFP	pXW23	MGSSGGSSGGSSGSKGEELFTGVVPILVELDGDVNGHKFSV RGEGEGDATNGKLTLKFICTTGKLPVPWPTLVTTLTYGVQC FSRYPDHMKRHDFFKSAMPEGYVQERTISFKDDGTYKTRAE VKFEGDTLVNRIELKGIDFKEDGNILGHKLEYNFNSHNVYIT ADKQKNGIKANFKIRHNVEDGSVQLADHYQQNTPIGDGPVL LPDNHYLSTQSVLSKDPNEKRDHMVLLEFVTAAGITHGMDE LYK
mCherry	pXW06	MVSKGEEDNMAIIKEFMRFKVHMEGSVNGHEFEIEGEGEGR PYEGTQTAKLKVTKGGPLPFAWDILSPQFMYGSKAYVKHPA DIPDYLKLSFPEGFKWERVMNFEDGGVVTVSQDSSLQDGEFI YKVKLRGTNFPSDGPVMQKKTMGWEASSERMYPEDGALK GEIKQRLKLKDGGHYDAEVKTTYKAKKPVQLPGAYNVNIK LDITSHNEDYTIVEQYERAEGRHSTGGMDELYK

LacZ (includes 6xHis tag)	pXW11	MTMITDSLAVVLQRRDWENPGVTQLNRLAAHPPFASWRNSEEARTDRPSQQLRSLNGEWRFAWFPAPEAVPESWLECDLPEADTVVVPSNWQMHGYDAPIYTNVTYPITVNPPFVPTENPTGCYSLTFNVDESWLQEGQTRIIFDGVNSAFHLWCNGRWVGYGQDSRLPSEFDLSAFLRAGENRLAVMVLRWSDGSYLEDQDMWRMSGIFRDVSLLHKPTTQISDFHVATRFNDDFSRAVLEAEVQMCGELRDYLRVTVSLWQGETQVASGTAPFGGEIIDERGGYADRVTLRLNVENPKLWSAEIPNLYRAVVELHTADGTLIEAEACDVGFREVRIENGLLLLNGKPLLIRGVNRHEHHPLHGQVMDEQTMVQDILLMKQNNFNAVRCSHYPNHPLWYTLCDRYGLYVVDEANIETHGMVPMNRLTDDPRWLPAMSERVTRMVQRDRNHPSVIIWSLGNESGHGANHDALYRWIKSVDPSRPVQYEGGGADTTATDIICPMYARVDEDQPFPAVPKWSIKKWLSLPGETRPLILCEYAHAMGNSLGGFAKYWQAFRQYPRLQGGFVWDWVDQSLIKYDENGNPWSAYGGDFGDTPNDRQFCMNGLVFADRTPHPALTEAKHQQFFQFRLSGQTIEVTSEYLFRHSDNELLHWMVALDGKPLASGEVPLDVAPQGKQLIELPELPQPESAGQLWLTVRVVQPNATAWSEAGHISAWQQWRLAENLSVTLPAASHAIPHLTTSEMDFCIELGNKRWQFNRQSGFLSQMWIGDKKQLLTPLRDQFTRAPLDNDIGVSEATRIDPNAWVERWKAAGHYQAEAALLQCTADTLADAVLITTAHAWQHQGKTLFISRKTYRIDGSGQMAITVDVEVASDTPHPARIGLNCQLAQVAERVNWLGLGPQENYPDRLTAACFDRWDLPLSDMYTPYVFPSENGLRCGTRELNYGPHQWRGDFQFNISRYSQQQLMETSHRHLLHAEEGTWLNIDGFHMGIGGDDSWSPSVSAEFQLSAGRYHYQLVWCQKHHHHHH
β-csn (includes 6xHis tag)	pXW13	MRELEELNVPGEIVESLSSSEESITRINKKIEKFQSEEQQQTEDELQDKIHPFAQTQSLVYPFPGPIPNSLPQNIPPLTQTPVVVPPFLQPEVMGVSKVKEAMAPKHKEMPFPKYPVEPFTESQSLTLTDVENLHLPLPLLQSWMHQPHQPLPPTVMFPPQSVLSLSQSKVLPVPQKAVPYPQRDMPIQAFLLYQEPVLGPVRGPFPIIVHHHHHH
Hmp	pXW20	MLDAQTIATVKATIPLLVETGPKLTAHFYDRMFTHNPELKEIFNMSNQRNGDQREALFNAIAAYASNIENLPALLPAVEKIAQKHTSFQIKPEQYNIVGEHLLATLDEMFSPGQEVLDAWGKAYGVLANVFINREAEIYNENASKAGGWEGTRDFRIVAKTPRSALITSFELEPVDGGAVAEYRPGQYLGVWLKPEGFPHQEIRQYSLTRKPDGKGYRIAVKREEGGQVSNWLHNHANVGDVVKLVAPAGDFFMAVADDTPVTLISAGVGQTPMLAMLDTLAKAGHTAQVNWFHAAENGDVHAFADEVKELGQSLPRFTAHTWYRQPSEADRAKGQFDSEGLMDLSKLEGAFSDPTMQFYLCGPVGFMQFTAKQLVDLGVKQENIHYECFGPHKVL
Hmp-sfGFP	pXW01	MLDAQTIATVKATIPLLVETGPKLTAHFYDRMFTHNPELKEIFNMSNQRNGDQREALFNAIAAYASNIENLPALLPAVEKIAQKHTSFQIKPEQYNIVGEHLLATLDEMFSPGQEVLDAWGKAYGVLANVFINREAEIYNENASKAGGWEGTRDFRIVAKTPRSA

		LITSFELEPVDGGAVAEYRPGQYLGVWLKPEGFPHQEIRQYS<br>LTRKPDGKGYRIAVKREEGGQVSNWLHNHANVGDVVKLV<br>APAGDFFMAVADDTPVTLISAGVGQTPMLAMLDTLAKAGH<br>TAQVNWFHAAENGDVHAFADEVKELGQSLPRFTAHTWYR<br>QPSEADRAKGQFDSEGLMDLSKLEGAFSDPTMQFYLCGPVG<br>FMQFTAKQLVDLGVKQENIHYECFGPHKVLGSSGGSSGGSS<br>GMSKGEELFTGVVPILVELDGDVNGHKFSVRGEGEGDATNG<br>KLTLKFICTTGKLPVPWPTLVTTLTYGVQCFSRYPDHMKRH<br>DFFKSAMPEGYVQERTISFKDDGTYKTRAEVKFEGDTLVNRI<br>ELKGIDFKEDGNILGHKLEYNFNSHNVYITADKQKNGIKANF<br>KIRHNVEDGSVQLADHYQQNTPIGDGPVLLPDNHYLSTQSV<br>LSKDPNEKRDHMVLLEFVTAAGITHGMDELYK
Hmp(Y29<br>F)-sfGFP	pXW14	MLDAQTIATVKATIPLLVETGPKLTAHFFDRMFTHNPELKEI<br>FNMSNQRNGDQREALFNAIAAYASNIENLPALLPAVEKIAQ<br>KHTSFQIKPEQYNIVGEHLLATLDEMFSPGQEVLDAWGKAY<br>GVLANVFINREAEIYNENASKAGGWEGTRDFRIVAKTPRSA<br>LITSFELEPVDGGAVAEYRPGQYLGVWLKPEGFPHQEIRQYS<br>LTRKPDGKGYRIAVKREEGGQVSNWLHNHANVGDVVKLV<br>APAGDFFMAVADDTPVTLISAGVGQTPMLAMLDTLAKAGH<br>TAQVNWFHAAENGDVHAFADEVKELGQSLPRFTAHTWYR<br>QPSEADRAKGQFDSEGLMDLSKLEGAFSDPTMQFYLCGPVG<br>FMQFTAKQLVDLGVKQENIHYECFGPHKVLGSSGGSSGGSS<br>GSKGEELFTGVVPILVELDGDVNGHKFSVRGEGEGDATNGK<br>LTLKFICTTGKLPVPWPTLVTTLTYGVQCFSRYPDHMKRHD<br>FFKSAMPEGYVQERTISFKDDGTYKTRAEVKFEGDTLVNRIE<br>LKGIDFKEDGNILGHKLEYNFNSHNVYITADKQKNGIKANF<br>KIRHNVEDGSVQLADHYQQNTPIGDGPVLLPDNHYLSTQSV<br>LSKDPNEKRDHMVLLEFVTAAGITHGMDELYK
Hmp-<br>sfGFP*	pXW04,<br>pXW08	MLDAQTIATVKATIPLLVETGPKLTAHFYDRMFTHNPELKEI<br>FNMSNQRNGDQREALFNAIAAYASNIENLPALLPAVEKIAQ<br>KHTSFQIKPEQYNIVGEHLLATLDEMFSPGQEVLDAWGKAY<br>GVLANVFINREAEIYNENASKAGGWEGTRDFRIVAKTPRSA<br>LITSFELEPVDGGAVAEYRPGQYLGVWLKPEGFPHQEIRQYS<br>LTRKPDGKGYRIAVKREEGGQVSNWLHNHANVGDVVKLV<br>APAGDFFMAVADDTPVTLISAGVGQTPMLAMLDTLAKAGH<br>TAQVNWFHAAENGDVHAFADEVKELGQSLPRFTAHTWYR<br>QPSEADRAKGQFDSEGLMDLSKLEGAFSDPTMQFYLCGPVG<br>FMQFTAKQLVDLGVKQENIHYECFGPHKVLGSSGGSSGGSS<br>GSKGEELFTGVVPILVELDGDVNGHKFSVRGEGEGDATNGK<br>LTLKFICTTGKLPVPWPTLVTTLTYGVQCFSRYPDHMKRHD<br>FFKSAMPEGYVQERTISFKDDGTYKTRAEVKFEGDTLVNRIE<br>LKGIDFKEDGNILGHKLEYNFNSHNVYITADKQKNGIKANF<br>KIRHNVEDGSVQLADHYQQNTPIGDGPVLLPDNHYLSTQSV<br>LSKDPNEKRDHMVLLEFVTAAGITHGMDELYK
Hmp-<br>mCherry	pXW05	MLDAQTIATVKATIPLLVETGPKLTAHFYDRMFTHNPELKEI<br>FNMSNQRNGDQREALFNAIAAYASNIENLPALLPAVEKIAQ

		KHTSFQIKPEQYNIVGEHLLATLDEMFSPGQEVLDAWGKAY<br>GVLANVFINREAEIYNENASKAGGWEGTRDFRIVAKTPRSA<br>LITSFELEPVDGGAVAEYRPGQYLGVWLKPEGFPHQEIRQYS<br>LTRKPDGKGYRIAVKREEGGQVSNWLHNHANVGDVVKLV<br>APAGDFFMAVADDTPVTLISAGVGQTPMLAMLDTLAKAGH<br>TAQVNWFHAAENGDVHAFADEVKELGQSLPRFTAHTWYR<br>QPSEADRAKGQFDSEGLMDLSKLEGAFSDPTMQFYLCGPVG<br>FMQFTAKQLVDLGVKQENIHYECFGPHKVLGSSGGSSGGSS<br>GMVSKGEEDNMAIIKEFMRFKVHMEGSVNGHEFEIEGEGEG<br>RPYEGTQTAKLKVTKGGPLPFAWDILSPQFMYGSKAYVKHP<br>ADIPDYLKLSFPEGFKWERVMNFEDGGVVTVSQDSSLQDGE<br>FIYKVKLRGTNFPSDGPVMQKKTMGWEASSERMYPEDGAL<br>KGEIKQRLKLKDGGHYDAEVKTTYKAKKPVQLPGAYNVNI<br>KLDITSHNEDYTIVEQYERAEGRHSTGGMDELYK
Hmp-mCherry*	pXW07	MLDAQTIATVKATIPLLVETGPKLTAHFYDRMFTHNPELKEI<br>FNMSNQRNGDQREALFNAIAAYASNIENLPALLPAVEKIAQ<br>KHTSFQIKPEQYNIVGEHLLATLDEMFSPGQEVLDAWGKAY<br>GVLANVFINREAEIYNENASKAGGWEGTRDFRIVAKTPRSA<br>LITSFELEPVDGGAVAEYRPGQYLGVWLKPEGFPHQEIRQYS<br>LTRKPDGKGYRIAVKREEGGQVSNWLHNHANVGDVVKLV<br>APAGDFFMAVADDTPVTLISAGVGQTPMLAMLDTLAKAGH<br>TAQVNWFHAAENGDVHAFADEVKELGQSLPRFTAHTWYR<br>QPSEADRAKGQFDSEGLMDLSKLEGAFSDPTMQFYLCGPVG<br>FMQFTAKQLVDLGVKQENIHYECFGPHKVLGSSGGSSGGSS<br>GVSKGEEDNMAIIKEFMRFKVHMEGSVNGHEFEIEGEGEGR<br>PYEGTQTAKLKVTKGGPLPFAWDILSPQFMYGSKAYVKHPA<br>DIPDYLKLSFPEGFKWERVMNFEDGGVVTVSQDSSLQDGEFI<br>YKVKLRGTNFPSDGPVMQKKTMGWEASSERMYPEDGALK<br>GEIKQRLKLKDGGHYDAEVKTTYKAKKPVQLPGAYNVNIK<br>LDITSHNEDYTIVEQYERAEGRHSTGGMDELYK
Hmp-LacZ (includes 6xHis tag)	pXW10	MLDAQTIATVKATIPLLVETGPKLTAHFYDRMFTHNPELKEI<br>FNMSNQRNGDQREALFNAIAAYASNIENLPALLPAVEKIAQ<br>KHTSFQIKPEQYNIVGEHLLATLDEMFSPGQEVLDAWGKAY<br>GVLANVFINREAEIYNENASKAGGWEGTRDFRIVAKTPRSA<br>LITSFELEPVDGGAVAEYRPGQYLGVWLKPEGFPHQEIRQYS<br>LTRKPDGKGYRIAVKREEGGQVSNWLHNHANVGDVVKLV<br>APAGDFFMAVADDTPVTLISAGVGQTPMLAMLDTLAKAGH<br>TAQVNWFHAAENGDVHAFADEVKELGQSLPRFTAHTWYR<br>QPSEADRAKGQFDSEGLMDLSKLEGAFSDPTMQFYLCGPVG<br>FMQFTAKQLVDLGVKQENIHYECFGPHKVLGSSGGSSGGSS<br>GTMITDSLAVVLQRRDWENPGVTQLNRLAAHPPFASWRNS<br>EEARTDRPSQQLRSLNGEWRFAWFPAPEAVPESWLECDLPE<br>ADTVVVPSNWQMHGYDAPIYTNVTYPITVNPPFVPTENPTG<br>CYSLTFNVDESWLQEGQTRIIFDGVNSAFHLWCNGRWVGY<br>GQDSRLPSEFDLSAFLRAGENRLAVMVLRWSDGSYLEDQD<br>MWRMSGIFRDVSLLHKPTTQISDFHVATRFNDDFSRAVLEA

		EVQMCGELRDYLRVTVSLWQGETQVASGTAPFGGEIIDERG GYADRVTLRLNVENPKLWSAEIPNLYRAVVELHTADGTLIE AEACDVGFREVRIENGLLLLNGKPLLIRGVNRHEHHPLHGQ VMDEQTMVQDILLMKQNNFNAVRCSHYPNHPLWYTLCDR YGLYVVDEANIETHGMVPMNRLTDDPRWLPAMSERVTRM VQRDRNHPSVIIWSLGNESGHGANHDALYRWIKSVDPSRPV QYEGGGADTTATDIICPMYARVDEDQPFPAVPKWSIKKWLS LPGETRPLILCEYAHAMGNSLGGFAKYWQAFRQYPRLQGGF VWDWVDQSLIKYDENGNPWSAYGGDFGDTPNDRQFCMNG LVFADRTPHPALTEAKHQQFFQFRLSGQTIEVTSEYLFRHS DNELLHWMVALDGKPLASGEVPLDVAPQGKQLIELPELPQP ESAGQLWLTVRVVQPNATAWSEAGHISAWQQWRLAENLS VTLPAASHAIPHLTTSEMDFCIELGNKRWQFNRQSGFLSQM WIGDKKQLLTPLRDQFTRAPLDNDIGVSEATRIDPNAWVER WKAAGHYQAEAALLQCTADTLADAVLITTAHAWQHQGKT LFISRKTYRIDGSGQMAITVDVEVASDTPHPARIGLNCQLAQ VAERVNWLGLGPQENYPDRLTAACFDRWDLPLSDMYTPYV FPSENGLRCGTRELNYGPHQWRGDFQFNISRYSQQQLMETS HRHLLHAEEGTWLNIDGFHMGIGGDDSWSPSVSAEFQLSAG RYHYQLVWCQKHHHHHH
Hmp-β-csn (includes 6xHis tag)	pXW12	MLDAQTIATVKATIPLLVETGPKLTAHFYDRMFTHNPELKEI FNMSNQRNGDQREALFNAIAAYASNIENLPALLPAVEKIAQ KHTSFQIKPEQYNIVGEHLLATLDEMFSPGQEVLDAWGKAY GVLANVFINREAEIYNENASKAGGWEGTRDFRIVAKTPRSA LITSFELEPVDGGAVAEYRPGQYLGVWLKPEGFPHQEIRQYS LTRKPDGKGYRIAVKREEGGQVSNWLHNHANVGDVVKLV APAGDFFMAVADDTPVTLISAGVGQTPMLAMLDTLAKAGH TAQVNWFHAAENGDVHAFADEVKELGQSLPRFTAHTWYR QPSEADRAKGQFDSEGLMDLSKLEGAFSDPTMQFYLCGPVG FMQFTAKQLVDLGVKQENIHYECFGPHKVLGSSGGSSGGSS GDDDDKRELEELNVPGEIVESLSSSEESITRINKKIEKFQSEEQ QQTEDELQDKIHPFAQTQSLVYPFPGPIPNSLPQNIPPLTQTPV VVPPFLQPEVMGVSKVKEAMAPKHKEMPFPKYPVEPFTESQ SLTLTDVENLHLPLPLLQSWMHQPHQPLPPTVMFPPQSVLSL SQSKVLPVPQKAVPYPQRDMPIQAFLLYQEPVLGPVRGPFPII VHHHHHH
sfGFP-Hmp	pXW15	MSKGEELFTGVVPILVELDGDVNGHKFSVRGEGEGDATNGK LTLKFICTTGKLPVPWPTLVTTLTYGVQCFSRYPDHMKRHD FFKSAMPEGYVQERTISFKDDGTYKTRAEVKFEGDTLVNRIE LKGIDFKEDGNILGHKLEYNFNSHNVYITADKQKNGIKANF KIRHNVEDGSVQLADHYQQNTPIGDGPVLLPDNHYLSTQSV LSKDPNEKRDHMVLLEFVTAAGITHGMDELYKGSSGGSSGG SSGLDAQTIATVKATIPLLVETGPKLTAHFYDRMFTHNPELK EIFNMSNQRNGDQREALFNAIAAYASNIENLPALLPAVEKIA QKHTSFQIKPEQYNIVGEHLLATLDEMFSPGQEVLDAWGKA YGVLANVFINREAEIYNENASKAGGWEGTRDFRIVAKTPRS

		ALITSFELEPVDGGAVAEYRPGQYLGVWLKPEGFPHQEIRQY<br>SLTRKPDGKGYRIAVKREEGGQVSNWLHNHANVGDVVKLV<br>APAGDFFMAVADDTPVTLISAGVGQTPMLAMLDTLAKAGH<br>TAQVNWFHAAENGDVHAFADEVKELGQSLPRFTAHTWYR<br>QPSEADRAKGQFDSEGLMDLSKLEGAFSDPTMQFYLCGPVG<br>FMQFTAKQLVDLGVKQENIHYECFGPHKVL
Hmp$_{25}$-<br>sfGFP	pXW16	MLDAQTIATVKATIPLLVETGPKLTGSSGGSSGGSSGSKGEE<br>LFTGVVPILVELDGDVNGHKFSVRGEGEGDATNGKLTLKFIC<br>TTGKLPVPWPTLVTTLTYGVQCFSRYPDHMKRHDFFKSAMP<br>EGYVQERTISFKDDGTYKTRAEVKFEGDTLVNRIELKGIDFK<br>EDGNILGHKLEYNFNSHNVYITADKQKNGIKANFKIRHNVE<br>DGSVQLADHYQQNTPIGDGPVLLPDNHYLSTQSVLSKDPNE<br>KRDHMVLLEFVTAAGITHGMDELYK
Hmp$_{100}$-<br>sfGFP	pXW17	MLDAQTIATVKATIPLLVETGPKLTAHFYDRMFTHNPELKEI<br>FNMSNQRNGDQREALFNAIAAYASNIENLPALLPAVEKIAQ<br>KHTSFQIKPEQYNIVGEGSSGGSSGGSSGSKGEELFTGVVPIL<br>VELDGDVNGHKFSVRGEGEGDATNGKLTLKFICTTGKLPVP<br>WPTLVTTLTYGVQCFSRYPDHMKRHDFFKSAMPEGYVQER<br>TISFKDDGTYKTRAEVKFEGDTLVNRIELKGIDFKEDGNILG<br>HKLEYNFNSHNVYITADKQKNGIKANFKIRHNVEDGSVQLA<br>DHYQQNTPIGDGPVLLPDNHYLSTQSVLSKDPNEKRDHMVL<br>LEFVTAAGITHGMDELYK
Hmp$_{200}$-<br>sfGFP	pXW18	MLDAQTIATVKATIPLLVETGPKLTAHFYDRMFTHNPELKEI<br>FNMSNQRNGDQREALFNAIAAYASNIENLPALLPAVEKIAQ<br>KHTSFQIKPEQYNIVGEHLLATLDEMFSPGQEVLDAWGKAY<br>GVLANVFINREAEIYNENASKAGGWEGTRDFRIVAKTPRSA<br>LITSFELEPVDGGAVAEYRPGQYLGVWLKPEGFPHGSSGGSS<br>GGSSGSKGEELFTGVVPILVELDGDVNGHKFSVRGEGEGDA<br>TNGKLTLKFICTTGKLPVPWPTLVTTLTYGVQCFSRYPDHM<br>KRHDFFKSAMPEGYVQERTISFKDDGTYKTRAEVKFEGDTL<br>VNRIELKGIDFKEDGNILGHKLEYNFNSHNVYITADKQKNGI<br>KANFKIRHNVEDGSVQLADHYQQNTPIGDGPVLLPDNHYLS<br>TQSVLSKDPNEKRDHMVLLEFVTAAGITHGMDELYK
Hmp$_{300}$-<br>sfGFP	pXW19	MLDAQTIATVKATIPLLVETGPKLTAHFYDRMFTHNPELKEI<br>FNMSNQRNGDQREALFNAIAAYASNIENLPALLPAVEKIAQ<br>KHTSFQIKPEQYNIVGEHLLATLDEMFSPGQEVLDAWGKAY<br>GVLANVFINREAEIYNENASKAGGWEGTRDFRIVAKTPRSA<br>LITSFELEPVDGGAVAEYRPGQYLGVWLKPEGFPHQEIRQYS<br>LTRKPDGKGYRIAVKREEGGQVSNWLHNHANVGDVVKLV<br>APAGDFFMAVADDTPVTLISAGVGQTPMLAMLDTLAKAGH<br>TAQVNWFHAAENGDGSSGGSSGGSSGSKGEELFTGVVPILV<br>ELDGDVNGHKFSVRGEGEGDATNGKLTLKFICTTGKLPVPW<br>PTLVTTLTYGVQCFSRYPDHMKRHDFFKSAMPEGYVQERTI<br>SFKDDGTYKTRAEVKFEGDTLVNRIELKGIDFKEDGNILGHK<br>LEYNFNSHNVYITADKQKNGIKANFKIRHNVEDGSVQLADH

		YQQNTPIGDGPVLLPDNHYLSTQSVLSKDPNEKRDHMVLLE<br>FVTAAGITHGMDELYK
LacZ-sfGFP	pXW22	MTMITDSLAVVLQRRDWENPGVTQLNRLAAHPPFASWRNS<br>EEARTDRPSQQLRSLNGEWRFAWFPAPEAVPESWLECDLPE<br>ADTVVVPSNWQMHGYDAPIYTNVTYPITVNPPFVPTENPTG<br>CYSLTFNVDESWLQEGQTRIIFDGVNSAFHLWCNGRWVGY<br>GQDSRLPSEFDLSAFLRAGENRLAVMVLRWSDGSYLEDQD<br>MWRMSGIFRDVSLLHKPTTQISDFHVATRFNDDFSRAVLEA<br>EVQMCGELRDYLRVTVSLWQGETQVASGTAPFGGEIIDERG<br>GYADRVTLRLNVENPKLWSAEIPNLYRAVVELHTADGTLIE<br>AEACDVGFREVRIENGLLLLNGKPLLIRGVNRHEHHPLHGQ<br>VMDEQTMVQDILLMKQNNFNAVRCSHYPNHPLWYTLCDR<br>YGLYVVDEANIETHGMVPMNRLTDDPRWLPAMSERVTRM<br>VQRDRNHPSVIIWSLGNESGHGANHDALYRWIKSVDPSRPV<br>QYEGGGADTTATDIICPMYARVDEDQPFPAVPKWSIKKWLS<br>LPGETRPLILCEYAHAMGNSLGGFAKYWQAFRQYPRLQGGF<br>VWDWVDQSLIKYDENGNPWSAYGGDFGDTPNDRQFCMNG<br>LVFADRTPHPALTEAKHQQQFFQFRLSGQTIEVTSEYLFRHS<br>DNELLHWMVALDGKPLASGEVPLDVAPQGKQLIELPELPQP<br>ESAGQLWLTVRVVQPNATAWSEAGHISAWQQWRLAENLS<br>VTLPAASHAIPHLTTSEMDFCIELGNKRWQFNRQSGFLSQM<br>WIGDKKQLLTPLRDQFTRAPLDNDIGVSEATRIDPNAWVER<br>WKAAGHYQAEAALLQCTADTLADAVLITTAHAWQHQGKT<br>LFISRKTYRIDGSGQMAITVDVEVASDTPHPARIGLNCQLAQ<br>VAERVNWLGLGPQENYPDRLTAACFDRWDLPLSDMYTPYV<br>FPSENGLRCGTRELNYGPHQWRGDFQFNISRYSQQQLMETS<br>HRHLLHAEEGTWLNIDGFHMGIGGDDSWSPSVSAEFQLSAG<br>RYHYQLVWCQKGSSGGSSGGSSGSKGEELFTGVVPILVELD<br>GDVNGHKFSVRGEGEGDATNGKLTLKFICTTGKLPVPWPTL<br>VTTLTYGVQCFSRYPDHMKRHDFFKSAMPEGYVQERTISFK<br>DDGTYKTRAEVKFEGDTLVNRIELKGIDFKEDGNILGHKLEY<br>NFNSHNVYITADKQKNGIKANFKIRHNVEDGSVQLADHYQQ<br>NTPIGDGPVLLPDNHYLSTQSVLSKDPNEKRDHMVLLEFVT<br>AAGITHGMDELYK

Green: sfGFP
Red: mCherry
Yellow: LacZ
Blue: Hmp (or truncated Hmp)
Purple: β-csn
GSSGGSSGGSSG: Linker
M: second start codon
HHHHHH: 6xHis tag
AANDENYALAA: ssrA tag
DDDDK: enterokinase cleavage site

References

29. Robinson, J.L.; Brynildsen, M.P. An ensemble-guided approach identifies ClpP as a major regulator of transcript levels in nitric oxide-stressed *Escherichia coli*. *Metab. Eng.* **2015**, *31*, 22-34. https://doi.org/10.1016/j.ymben.2015.06.005.
30. Baba, T.; Ara, T.; Hasegawa, M.; Takai, Y.; Okumura, Y.; Baba, M.; Datsenko, K.A.; Tomita, M.; Wanner, B.L.; Mori, H. Construction of *Escherichia coli* K-12 in-frame, single-gene knockout mutants: the Keio collection. *Mol. Syst. Biol.* **2006**, *2*, 2006.0008-2006.0008. https://doi.org/10.1038/msb4100050.
31. Orman, M.A.; Brynildsen, M.P. Dormancy Is Not Necessary or Sufficient for Bacterial Persistence. *Antimicrob. Agents Chemother.* **2013**, *57*, 3230-3239. https://doi.org/10.1128/AAC.00243-13.
32. Zaslaver, A.; Bren, A.; Ronen, M.; Itzkovitz, S.; Kikoin, I.; Shavit, S.; Liebermeister, W.; Surette, M.G.; Alon, U. A comprehensive library of fluorescent transcriptional reporters for *Escherichia coli*. *Nat. Methods* **2006**, *3*, 623-628. https://doi.org/10.1038/nmeth895.
33. Amato, S.M.; Brynildsen, M.P. Nutrient Transitions Are a Source of Persisters in Escherichia coli Biofilms. *PLoS ONE* **2014**, *9*, e93110. https://doi.org/10.1371/journal.pone.0093110.
34. Amato, S.M.; Brynildsen, M.P. Persister Heterogeneity Arising from a Single Metabolic Stress. *Curr.* **2015**, *25*, 2090-2098. https://doi.org/10.1016/j.cub.2015.06.034.
28. Wan, X.; Brynildsen, M.P. Robustness of nitric oxide detoxification to nitrogen starvation in *Escherichia coli* requires RelA. *Free Radic. Biol. Med.* **2021**, *176*, 286-297. https://doi.org/10.1016/j.freeradbiomed.2021.10.005.
35. Datsenko, K.A.; Wanner, B.L. One-step inactivation of chromosomal genes in *Escherichia coli* K-12 using PCR products. *Proc. Natl. Acad. Sci. U.S.A.* **2000**, *97*, 6640. https://doi.org/10.1073/pnas.120163297.
36. Cherepanov, P.P.; Wackernagel, W. Gene disruption in *Escherichia coli*: TcR and KmR cassettes with the option of Flp-catalyzed excision of the antibiotic-resistance determinant. *Gene* **1995**, *158*, 9-14. https://doi.org/10.1016/0378-1119(95)00193-A.

*Note that the reference number here is consistent with reference numbers in the main text.
